# Toward social-health integration in Sicily: description of the first hub and spoke model to improve the diagnostic therapeutic care paths for neurorehabilitation

**DOI:** 10.3389/fpubh.2023.1141581

**Published:** 2023-05-16

**Authors:** Maria Cristina De Cola, Augusto Ielo, Viviana Lo Buono, Angelo Quartarone, Rocco Salvatore Calabrò

**Affiliations:** IRCCS Centro Neurolesi Bonino Pulejo, Messina, Italy

**Keywords:** hub and spoke, organization design, neuro-rehabilitation, quality indicators, robotic device

## Abstract

**Introduction:**

The study describes a hub and spoke network for neuro-rehabilitation recently activated in Sicily, and evaluates the before-after changes yielded, in terms of integrated care.

**Methods:**

A set of indicators based on data contained in the administrative database of inpatients of the Regional Health System are presented and discussed. Statistical analysis was conducted both globally and separately for the 9 Sicilian provinces (Agrigento, Caltanissetta, Catania, Enna, Messina, Palermo, Siracusa, Ragusa, and Trapani).

**Results:**

Results showed an increase in admissions of people residing in the province where the Spokes have been opened: Trapani (+32.4%), Messina (+7.8%) and Palermo (+4.4%); besides a significant increase of patients from healthcare facilities proportion (*p* = 0.001) and from acute wards (*p* = 0.029). In addition, we found a decrease of discharge to protected healthcare facilities (*p* = 0.001) and to acute wards (*p* < 0.001), as well as an increase of discharges to home (*p* = 0.018).

**Discussion:**

In conclusion, it would seem that the activation of this network has facilitated the management of these patients, avoiding unnecessary migrations to other provinces and/or regions, and improving the regional care service for neuro-rehabilitation. Future research will be direct to investigate this aspect, focusing on before-after variations in hospitalization rates and origin– destination patient flows.

## 1. Introduction

According to the WHO principles of protecting worldwide population health, the Italian National Health Service (NHS) made social and health integration one of its pillars according to the Legislative Decree 229/1999. Since then, many steps forward have been made In Italy, both at national and local regional level. In Sicily a rehabilitation plan was designed in 2012 to deal with the lack of highly specialized rehabilitation services, as well as of an inadequate integration between hospital and territorial services (1). Indeed, the Sicilian government expressed its intention to requalify the rehabilitation facilities through the creation of a hub and spoke (HS) model, with the purpose of developing new guidelines, assistance protocols and recommendations on the rehabilitation pathway. A HS model includes a vertical organization with rules extending from the hub to the spokes in order to maximize efficiencies and effectiveness (2), and in the last years, many integrated models designed as HS networks emerged in several health areas, including the organization and delivery of novel rehabilitation services (3–7).

In 2017 Sicily introduced the first Regional Socio-Health Plan, which defines an integrated system of rules that brings together health and social care (8). It is an interdisciplinary care plan designed to respond to the organizational fragmentation of health services. Thus, territorial services and home care programs can guarantee a continuity between care and rehabilitation, social inclusion and job reintegration. The challenge is to provide better health care with significant cost reduction, i.e., reducing hospitalization, improving the patient quality of life of older adults, disabled, patients suffering from chronic (often degenerative) and rare diseases.

In this scenario a new Diagnostic, Therapeutic and Care Pathway (DTCP) emerged, since it can be considered a tool of governance through which the Region defines guidelines for care processes centered on the patient’s need, taking into account the resources available (9). Thus, health services delivered to citizens in a circular continuum approach can change the prognosis of certain diseases (10). DTCP for neurorehabilitation includes several healthcare professionals operating in different settings (primary, intermediate and hospital care) to provide better management by a multidisciplinary team in a redefinition of a model based on the continuity of care (11). An integrating neurorehabilitation service offers the opportunity to significantly improve patient outcomes, terms of residual disability and performance improvement (12). The DTCP for intensive and extensive neurorehabilitation is aimed at providing advice and guidelines about the management of neurological inpatients. In detail, intensive rehabilitation involves those patients in which the recovery could be maximized (e.g., those affected by stroke or traumatic brain injury), and then, if they do not have medical or psychiatric contraindications, the training should last at least 3 h per day whereas nursing assistance is provided on a 24 h basis. Extensive neurorehabilitation is instead provided 1–3 h per day, according to the patient’s condition and potential recovery. Usually, patients with severe acquired brain injury are labeled as cod 75 and require the higher degree of care and cure; patients with spinal cord injury (cod 28) are provided with the same intensive care as cod 75, whereas patients with stroke, multiple sclerosis and other orthopedic and cardiopulmonary diseases are considered as cod 56, where rehabilitation and nursing are less intensive.

Many studies showed that a HS model can be included within a DTCP to manage different stages of neurological diseases from the acute phase or disease diagnosis (13, 14) to the rehabilitation (15) and return to normal life. The strong point of the HS network is the possibility of avoiding unnecessary travel (since the local spokes may provide nearly the same high-quality services than the hub), by reducing costs both from the healthcare system and patient’s perspective, but improving the medical service offered (16, 17).

Thus, with the view of improving the social and health integration of the Sicilian DTCP for intensive neurorehabilitation within a territorial continuity path care, covering the unsatisfied demand for care and supporting the users within their own territory, a hub and spoke model was designed and has been tested for 5 years (since January 2017 to June 2022) by the IRCCS Centro Neurolesi “Bonino-Pulejo” of Messina, Sicily. The HS network defines the operating rules, the monitoring system, the quality and safety requirements of the processes and care paths, the qualification of professionals, and the ways of involving subjects. We assume that an integrated neurorehabilitation model might fully satisfy the patient care needs, providing interventions in compliance with the continuity of care process and clinical and organizational appropriateness.

The aim of this study is to describe in detail this Sicilian HS model and to evaluate the changes that occurred by its activation, especially in terms of integrated care. Indeed, we believe that a multidisciplinary and integrated care path dedicated to neurological patients, could led to better outcomes in terms of hospitalization and access to the rehabilitation network.

Thus, we compared the care paths of neurorehabilitation hospitalization patients in 2016 (i.e., before the introduction of the HS network) with the care paths of neurorehabilitation hospitalization patients in 2018 (after the introduction of the HS network and before the COVID-19 pandemic) through a set of suitably defined indicators.

## 2. The hub and spoke rehabilitation model

The IRCCS Centro Neurolesi “Bonino-Pulejo” of Messina is an experimental hub and spoke model to integrate the intensive neurorehabilitation process within a territorial DTCPs. This HS model derives from a regional project, designed in collaboration with the Ministry of Health and the Sicilian Government, which includes a network of Rehabilitation Units (Spokes) located throughout the Sicily, under the coordination of a Hub Centre (i.e., the IRCCS Centro Neurolesi). All patients treated in this HS presented neurological impairment due to stroke, spinal cord injury, brain injury and neurodegenerative diseases (e.g., Parkinson’s disease or multiple sclerosis). To provide the best rehabilitation activities possible, each spoke center was equipped with innovative technologies for basic robotic rehabilitation (i.e., Lokomat, Erigo, Armeo power, VRRS, Pegaso Ciclo-FES), in addition to a gym where they performed conventional physiotherapy. Moreover, with the purpose to facilitate the empowerment of the disabled people, besides physiotherapy, any center provides speech therapy, neuropsychological rehabilitation, occupational therapy, psychological support to both patients and caregivers and social welfare assistance. Therefore, patients had access to the most modern treatments to recover functions, improve the quality of life, and facilitate the social reintegration at discharge.

**Figure 1:**
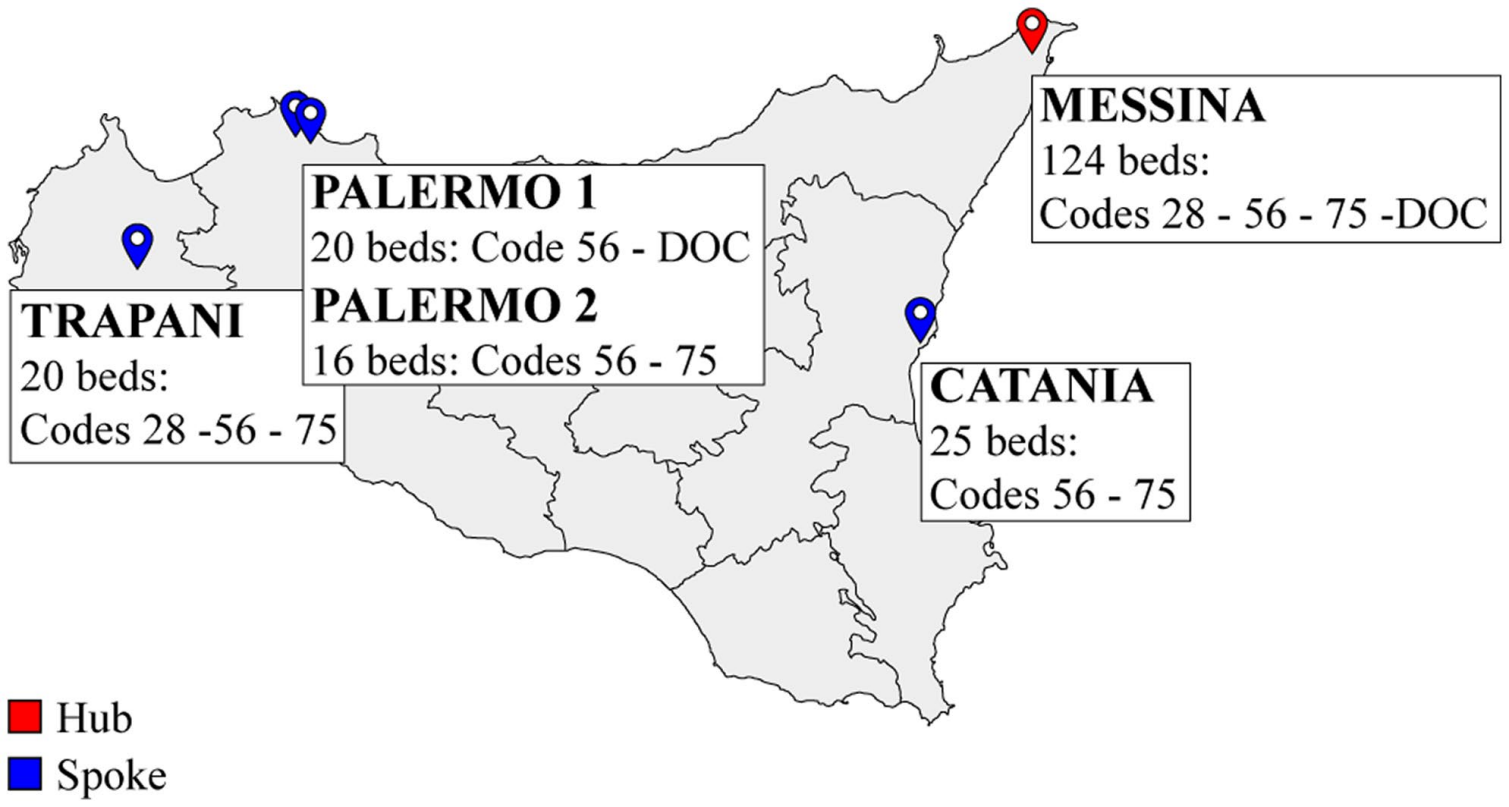
Geographical representation of the hub and spoke rehabilitation network in Sicily. DOC, disorder of consciousness.

Throughout 2017, the first three spoke centers were opened: one in Trapani (January) and two in Palermo (one in August and the other in November) leading to 56 new beds to supply the demand for neurological rehabilitation services in the west of the Region. In July 2018, the fourth center was placed in Catania with 25 new beds. Patients admitted to some spoke centers differ in severity, as well as in diagnosis, as shown in Figure 1.

### 2.1. Inpatient admission

Patients admission to each spoke was established by the hub center. Clinicians and or families in charge of the patients had to send a request for admission to the hub center by attaching the clinical discharge report from the acute department or the clinical report of a recent outpatient view. A specific hospital commission including neurologists and physiatrists evaluated the documentation and assigned a score based on individual clinical conditions, placing the patient in the ranking of the qualified spoke center. If the patient was not so severely affected, he/she was admitted to the spoke placed next to the residence area.

### 2.2. Supplies

The hub center has neurophysiology, neuroimaging and diagnostic labs, physiotherapy gym, advanced robotic and virtual reality devices, and telemedicine services after discharge (for more details see Table 1). On the contrary, the spoke centers only have physiotherapy gym and basic robotic devices and virtual reality. However, when necessary, neurorehabilitation specialists use telemedicine to improve services and provide a second opinion. Moreover, specialists of the hub center carried out interventions at each spoke center, such as pneumology, otorhinolaryngology, nutrition, endocrinology and neurophysiology. Other counseling activities, as well as neuroimaging and diagnostic evaluations, could be provided by the local healthcare services (ASP) whether necessary.

**Table 1:** Comparative analysis by citizen’s province of residence - rehabilitation wards (units 28/56/75).

Province	Agrigento	Caltanissetta	Catania	Enna	Messina	Palermo	Ragusa	Siracusa	Trapani	Sicily
**2016**										
N. Hospitalizations	288	238	830	214	456	558	203	229	166	3,182
Length of stay (AVG)	56.6 ± 41.7	52.4 ± 49.6	50.2 ± 42.9	101.6 ± 90.9	56.5 ± 51.3	67.0 ± 59.2	47.8 ± 49.3	47.6 ± 34.9	65.8 ± 54.0	58.7 ± 54.2
HwP (%)	64.6	46.2	73.3	73.8	82.0	66.7	68.5	59.4	46.4	67.9
LwP (AVG)	34.9 ± 40.1	14.8 ± 19.1	31.3 ± 29.4	87.3 ± 99.0	42.1 ± 50.1	41.0 ± 54.2	23.7 ± 23.8	20.7 ± 21.5	21.4 ± 26.2	35.7 ± 47.9
Age (AVG)	61.1 ± 17.0	60.4 ± 18.6	65.6 ± 16.9	58.0 ± 16.7	63.1 ± 17.6	58.6 ± 17.1	64.4 ± 17.5	63.8 ± 16.8	64.5 ± 13.6	62.4 ± 17.2
Over 75 (%)	21.9	29.8	36.3	22.4	29.6	17.0	33.0	34.1	24.1	28.2
Women (%)	45.1	51.7	51.0	68.2	47.1	44.1	43.3	46.3	46.4	48.8
Acute in (%)	5.6	8.4	15.3	2.3	18.9	21.1	11.8	13.1	19.3	14.4
Home in (%)	74.3	74.8	38.9	77.6	66.2	62.5	56.2	36.2	62	57.6
Other in (%)	6.2	13.4	48.2	8.9	17.1	21.5	32.5	31.4	9.6	25.8
Waiting time (AVG)	20.6 ± 47.6	30.3 ± 63.9	15.7 ± 43.5	31.0 ± 55.9	21.7 ± 57.0	15.8 ± 46.6	18.0 ± 55.9	13.8 ± 47.8	11.5 ± 28.9	18.9 ± 49.9
Protected out (%)	18.8	5.9	9.8	7.5	8.6	10.4	10.8	20.5	15.7	11.2
Acute out (%)	5.6	6.7	12.8	4.7	11.6	9.7	14.3	7.4	7.2	9.8
Home out (%)	69.4	82.4	72.7	81.8	76.3	68.8	70.9	66.4	74.7	73.1
**2018**										
N. Hospitalizations	315	226	927	186	821	682	159	299	290	3,905
Length of stay (AVG)	60.8 ± 47.9	51.6 ± 49.5	52.9 ± 49.2	104.7 ± 100.5	56.2 ± 56.2	71.4 ± 65.8	48.1 ± 43.9	47.4 ± 45.4	62.2 ± 47.1	59.9 ± 57.9
HwP (%)	67.9	38.1	68.6*	69.9	88.9***	69.8	61.0	55.9	68.6***	70
LwP (AVG)	37.7 ± 41.4	12.4 ± 18.2	30.2 ± 32.4	86.2 ± 107.6	45.6 ± 52.4	48.6 ± 65.5*	21.8 ± 24.8	19.9 ± 21.9	35.7 ± 30.9***	38.2 ± 51.4*
Age (AVG)	61.7 ± 15.6	60.4 ± 18.0	65.8 ± 16.9	59.6 ± 16.6	65.7 ± 16.0**	59.4 ± 16.4	64.0 ± 17.0	63.2 ± 16.3	62.5 ± 14.9	63.2 ± 16.6
Over 75 (%)	20.6	23.5	35.5	23.7	32.3	18.0	32.7	25.4*	22.4	27.5
Women (%)	53	52.7	49	65.1	47.1	43	39	43.8	44.5	47.7
Acute in (%)	7.6	12.4	16.5	4.3	22.4	20.5	13.2	12.0	14.8	16.3*
Home in (%)	90.8***	71.7	35.9	78.0	69.5	59.1	45.3	42.8	59.3	58.2
Other in (%)	3.5	21.2*	52.8	12.4	18.3	24.8	42.8	33.1	30.0***	29.3**
Waiting time (AVG)	24.3 ± 47.7	23.1 ± 45.7	18.4 ± 51.5	22.2 ± 39.0	22.0 ± 57.9	24.6 ± 45.9***	19.4 ± 55.9	19.8 ± 51.0	24.7 ± 43.5***	21.8 ± 50.5*
Protected out (%)	20.3	9.7	13.5*	4.3	8.9	10.4	15.7	15.4	6.9**	11.6
Acute out (%)	6.0	4.0	11.8	2.2	3.8***	6.9	3.1***	5.0	7.2	6.7***
Home out (%)	69.5	79.2	70.9	84.4	82.0*	73.3	74.8	73.2	79.0	75.6*

Quantitative variables are expresses as mean ± standard deviation; HwP = Proportion of hospitalizations in facilities placed within the patients’ own province of residence; LwP = Average length of stay in facilities placed within the patients’ own province of residence. *p < 0.05; **p < 0.01; ***p < 0.001.

### 2.3. Staff training

The high-skilled personnel of the hub center trained the spoke’s healthcare staff to apply the appropriate DTCP as well as correctly manage and use the robotic devices. Moreover, the hub center constantly supports the rehabilitation team of the spoke centers in order to ensure high standards of patient’s management through the use of telemedicine. Indeed, the Virtual Reality Rehabilitation System (VRRS) can be also used real-time to help the Spokes’s healthcare professionals providing rehabilitation to their inpatients (18, 19). A weekly evaluation of the individual rehabilitation plans and consultations about the current problems were also provided.

## 3. Methods

### 3.1. Data source

This study gathered information from the inpatient administrative database of the Regional Health System (RHS) from January 2016 to December 2018, including all discharges from rehabilitation/neurology wards in Sicily, as well as those of Sicilian residents that occurred in other Italian Regions. The hospitalizations studied for comparison were only those from the years 2016 and 2018; hospitalizations from the year 2017 were excluded. To identify hospitalizations, we used the version 24 of the diagnosis-related group codes (DRG) of the International Classification of Diseases, 9th Revision, Clinical Modification (ICD-9-CM) (20), and we selected only hospitalization with a DRG codes belonging to the Major Diagnostic Category 1 (Diseases and Disorders of the Nervous System - MDC 1). We also filtered for clinical specialty and hospital disciplines codes, defined by the Italian Health Ministry (21): code 28 (spinal cord unit), 56 (functional recovery and rehabilitation) and 75 (neurorehabilitation; DOC: disorder of consciousness) for rehabilitation wards, whereas 32 (neurology) and 49 (intensive unit) for acute wards were also evaluated to ascertain their transfer in a rehabilitation unit. Hospitalizations of non-Sicilian residents as well as pediatric patients were excluded. Voluntary discharge of the patient (against the advice of a doctor) were also excluded. To guarantee the quality of the data and the reliability of the results, we have selected from the database only complete records, i.e., records including patient’s demographic characteristics, spatial variables (region and province), and information concerning the hospitalization.

### 3.2. Outcome measures

First of all, we examined the variation of the distribution of the hospitalizations within each province and the average length of stay (LOS), comparing the years 2016 and 2018. The proportion of hospitalizations in facilities placed within the patients’ own province of residence (HwP), as well as the mean duration of these hospitalizations (LwP), have been considered a measure of demand satisfied by the province. Second, we sought changes in the demographic structure of the patients hospitalized. We then computed the average age, the percentage of patients over 75, and the percentage of women by province. Finally, to understand the role that the HS network could have on the DTCP, we observed: (i) variations in the access to hospital care by the proportion of patients coming from an acute ward (Acute in), from home (Home in), or from a different (public or private) healthcare facility (Other in), the average waiting time for hospitalization (Waiting time); (ii) variations in discharge outcomes by the proportion of discharges to home (Home out), protected discharges (Protected out), i.e., patients discharged to a nursing home or patients receiving home health care after being discharged, and transfers to an acute ward (Acute out).

### 3.3. Statistical analysis

The analysis was conducted both globally and separately by the 9 Sicilian provinces (Agrigento, Caltanissetta, Catania, Enna, Messina, Palermo, Siracusa, Ragusa, and Trapani).

We compared the above measures before and after the HS network activation, i.e., 2016 versus 2018. Statistical analysis was performed by using the 4.2.2 version of the open-source software R. A *p* < 0.05 was considered as statistically significant. Results for continuous variables were expressed in mean ± standard deviation, whereas categorical variables in frequencies and percentages. The Chi-square test with continuity correction was used to assess for statistical differences in proportions, whereas the unpaired Student’s *t*-test was used to compare continuous variables.

## 4. Results

A total of 7,087 hospitalization records in rehabilitation units were included in this study. Table 1 reports results of the comparative analysis performed on the study outcome measures.

### 4.1. Demographic structure

The mean age of the patients hospitalized in 2016 was 62.4 ± 17.2 years, whereas in 2018 was 63.2 ± 16.6 years. Overall, no significant 2018-2016 changes in proportions for either gender or age (over 75) were found. However, the mean age resulted to be statistically increased (*t*(341) = 2.18, *p* = 0.03) in patients attending spinal cord unit (discipline code 28), with a higher proportion of over 75 in 2018 than in 2016 (+42.5%).

### 4.2. Hospitalizations and length of stay

There was no significant change in the overall mean length of stay between 2016 and 2018 (*t*(6951) = 0.91; *p* = 0.362). After the establishment of the HS network, we observed an increase in HwP in the provinces of Trapani (+32.4%), Messina (+7.8%) and Palermo (+4.4%). In the same provinces we also observed an increase in LwP: Trapani (+40.1%), Messina (+7.7%) and Palermo (+15.6%).

**Figure 2:**
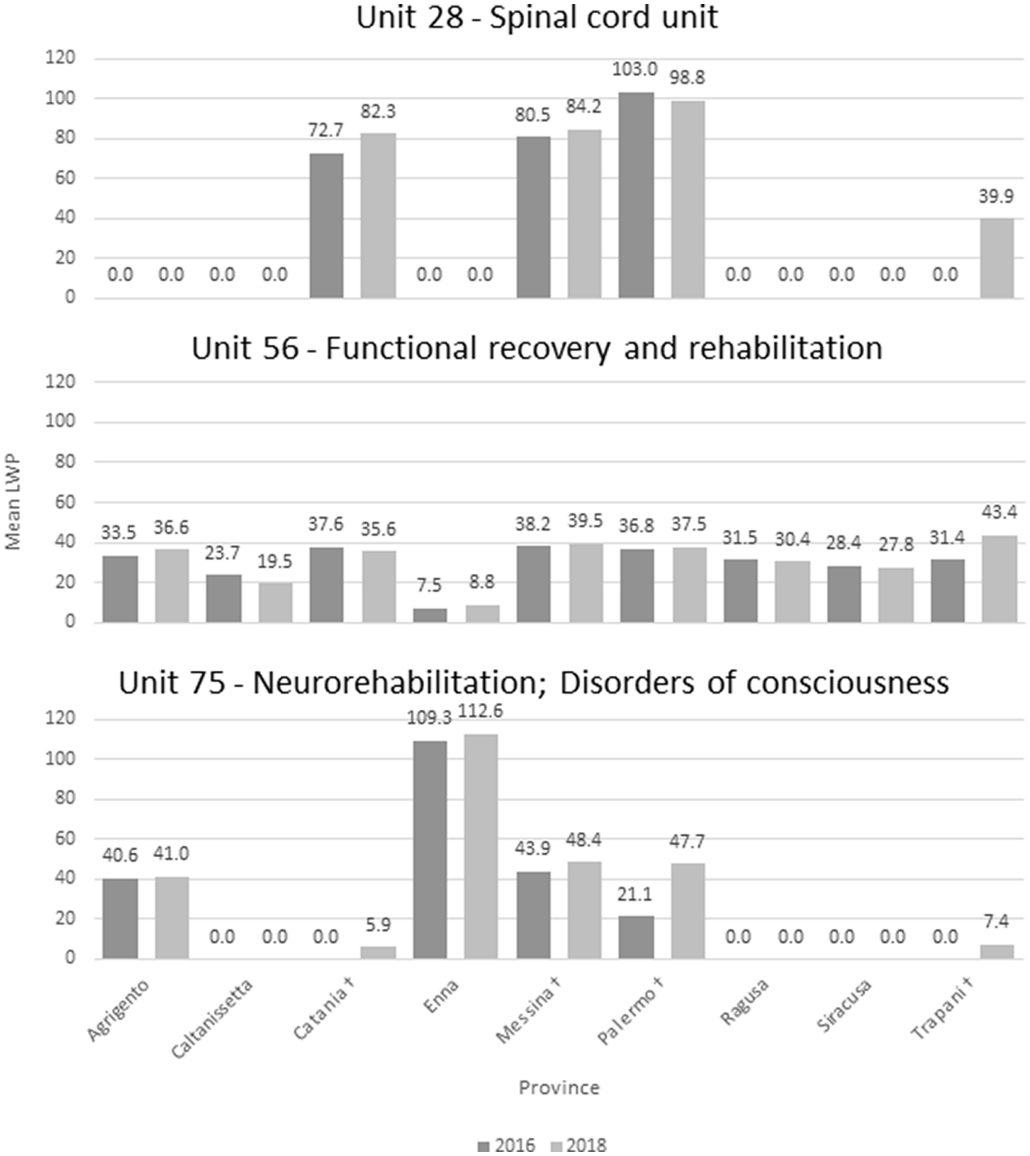
Mean LWP by rehabilitation ward (units 28/56/75) and province. ^†^Province included in the HS network.

Figure 2 shows a detailed description of the LwP indicator by rehabilitation ward and province. Statistically significant changes between the 2 years were reported within the unit/code 28 and 56 in the province of Trapani (*t*(19) = 4.5, *p* < 0.001; *t*(226) = 3.90, *p* < 0.001, respectively), and also within the unit/code 75 in the province of Catania (+100%), Palermo (+55.8%) and Trapani (+100%).

### 4.3. Access to hospital care

**Figure 3:**
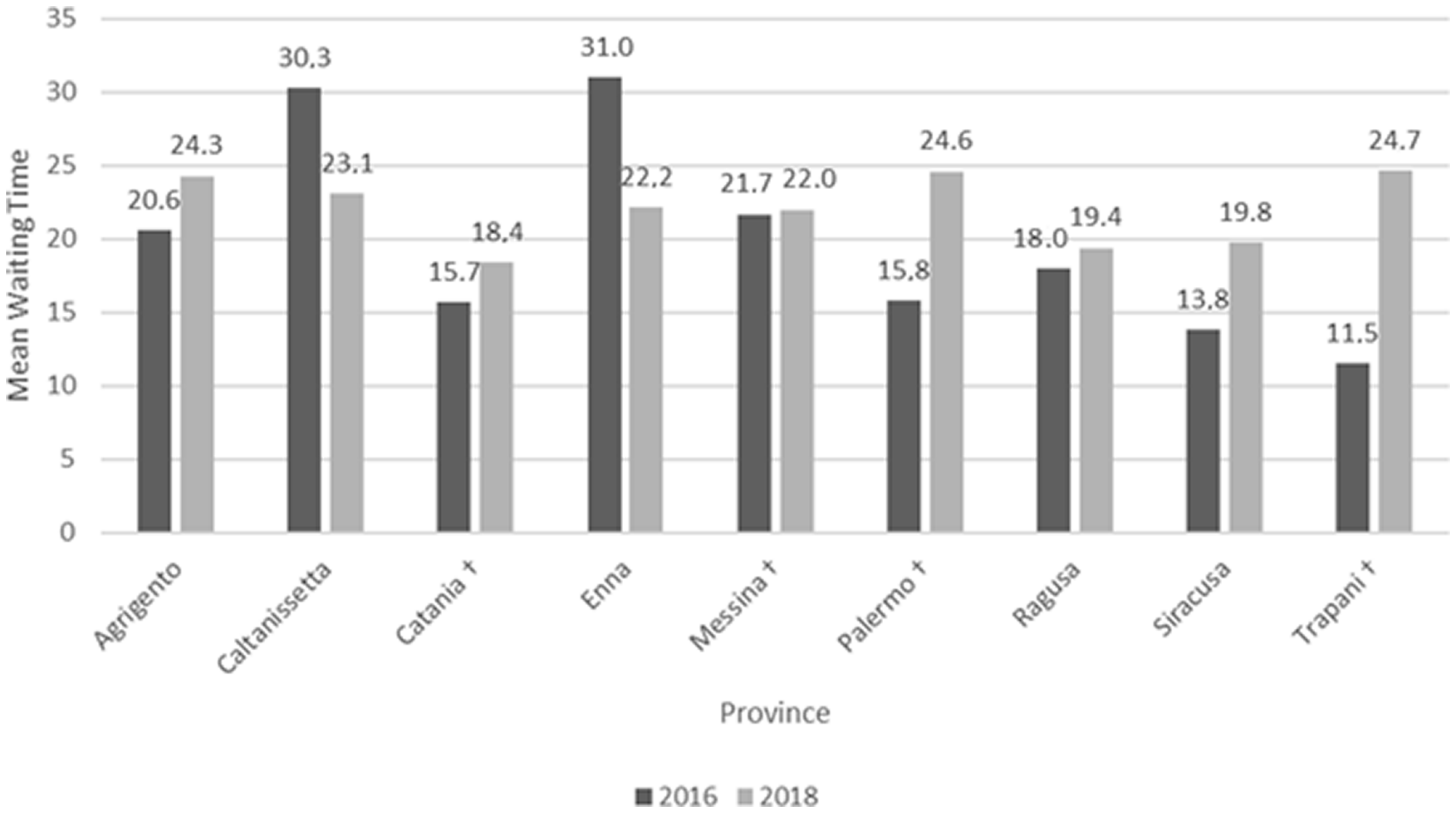
Mean waiting time for hospitalization by province. ^†^Province included in the HS network.

Overall, we observed a significant increase of *Acute in* proportion (*χ*^2^ (1) = 4.79, *p* = 0.029) from 2016 to 2018, whereas there was no significant change of *Home in* proportion (*χ*^2^ (1) = 0.24, *p* = 0.623). In addition, we found a statistically significant increase of *Other in* proportion (*χ*^2^ (1) = 10.5, *p* = 0.001) between 2016 and 2018. As shown in Figure 3, we observed a statistically significant increase of *Waiting time* in the provinces of Palermo (*t*(1182) = 3.32, *p* = 0.001) and Trapani (*t*(444) = 3.86, *p* < 0.001). On the contrary, in the provinces of Catania and Messina we observed an increase (from 15.7 ± 43.5 to 18.4 ± 51.5; from 21.7 ± 57.0 to 22.0 ± 57.9, respectively) that did not reach the statistical significance (*t*(1748) = 1.21, *p* = 0.225; *t*(952) = 0.10, *p* = 0.919, respectively).

### 4.4. Discharge outcomes

**Figure 4:**
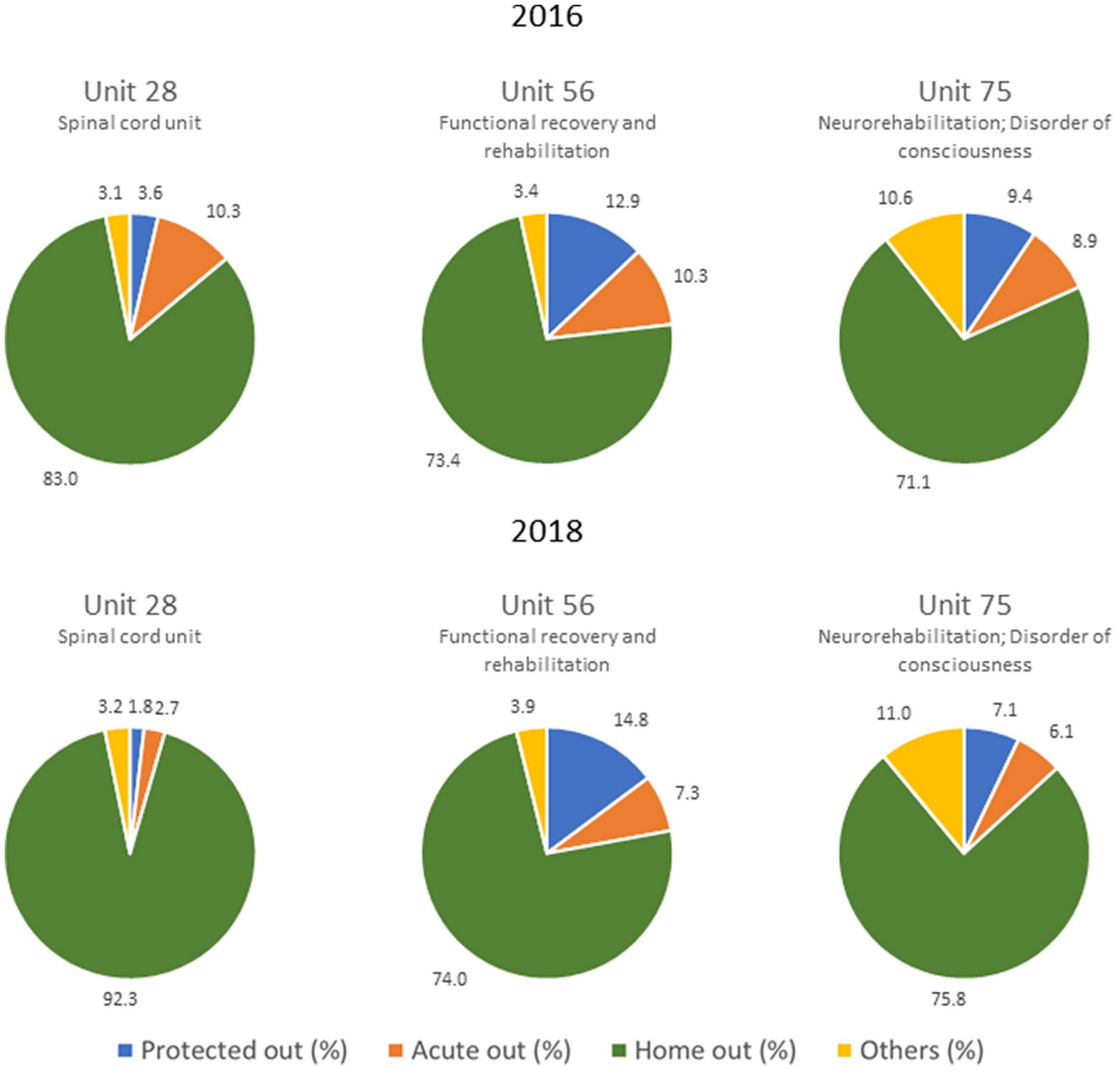
Discharge outcomes by rehabilitation wards and year.

Overall, between 2016 and 2018, there was a statistically significant reduction of *Acute out* proportion (*χ*^2^ (1) = 23.41, *p* < 0.001), and a significant increase of *Home out* (*χ*^2^ (1) = 5.62, *p* = 0.018). On the contrary, there was no significant change in *Protected out* proportion (*χ*^2^ (1) = 0.25, *p* = 0.619) from 2016 to 2018. We found similar results when performing 2018–2016 comparisons differencing by rehabilitation ward, as shown in Figure 4. Significant changes in *Acute out* emerged within the spinal unit (code 28) and the neurorehabilitation unit (code 75): *χ*^2^ (1) = 8.33, *p* = 0.004; *χ*^2^ (1) = 6.18, *p* = 0.013, respectively; as well as in *Home out*: *χ*^2^ (1) = 6.93, *p* = 0.008; *χ*^2^ (1) = 6.60, *p* = 0.01, respectively.

## 5. Discussion

This is the first Italian HS network for intensive neurorehabilitation that sought to improve quality of care and reduce patient migration (22). Indeed, to face the demand variation for care, the Sicilian government is heading toward a people-centered process of health services, reinforcing the existing DTCPs in order to support the clients in their own territory, and involving the general practitioners (GP) as consultants. After all, citizens identify their GP as the preferred point of access to local health services compared to other health professionals (23, 24). In this perspective, GP can collaborate with facilities of the RHS in an integrated network, giving a central role to the patient (25).

In this study, we focused on the evaluation of effects that this HS model had on the improvement of the health service and in hospitalization outcomes, as well as the integration and accessibility to the services, using a set of indicators specifically designed. Indeed, the integration of procedures among the various clinical settings requires careful monitoring of both the resources used and the results achieved in the different phases of the care process. Such monitoring, together with a critical assessment of the innovative health policies adopted can be a useful tool for supporting both the planning and the sustainability of the RHS. For example, a HS model can allow to avoid unnecessary migrations to other provinces and/or regions, with a consequent reduction of hospitalizations costs (26). This is exactly what we observed in our case: there has been an increase in admissions and average length of stay of people residing in the province where the Spokes have been opened. In addition, our findings show a significant increase in the spoke admission of patients from other care facilities and from acute wards, which suggests that the opening of spokes could have (i) attracted a portion of patients who were previously served by private facilities and/or services, and (ii) improved RHS continuity of care service, with regard to the possibility for acute hospital wards to discharge to the new dedicated beds of the rehabilitation spokes facilities introduced in the provinces of Sicily (Acute in).

Indeed, before the application of our model, many patients were forced to move to other regions or out of Italy (e.g., Austria) where the rehabilitation pathway is more advanced and integrated to the territorial services. In Northern Italy, for example, patients with severe traumatic brain injury have a “continuity of care” network, called GRACER, for their complete management from the acute phase to the return home/to work. Patients and their caregivers are totally assisted since the admission to Intensive Care, during their stay in Neurorehabilitation Unit, their training as outpatients (Day-H and ambulatory clinics) and then also at home. Moreover, territorial services and No-profit associations help them (when the clinical condition allow this) in their social-economic life (e.g., to find job, practice a sport, etc.)

Results also show a decrease of discharge to protected healthcare facilities and to acute wards, as well as an increase of discharges to home. It could be hypothesized that the high quality of rehabilitation services provided by the Spokes (also thanks to the continuous supervision by the hub) may have led to better outcomes (although no specific clinical outcomes have been assessed) further improving the quality of the DTCP.

Therefore, to define a battery of indicators able to monitor the DTCP’s outcomes in a standard way for all RHS facilities is becoming necessary, because of the recent growth of hospitalizations for intensive neurorehabilitation, especially for older adults (27). The increase of life expectancy makes the aging a risk factor for the development of multiple chronic diseases, including cardiovascular, cerebrovascular and neurodegenerative disorders (28), causing a significant economic burden for the healthcare system (29). Our findings report an increase of the mean age of the hospitalized patients, and of the over 75 proportion, concerning the spinal unit patients (code 28): this sustains the hypothesis that the opening of a spoke facilitates the management of these patients within the family, avoiding unnecessary displacement of these frail patients.

As a main strength, this model was conceived to properly address the issues related to neurorehabilitation in the main and underserved provinces of Sicily. Indeed, besides the specialistic rehabilitative management of acquired brain and spinal cord injury, the HS model provided rehabilitation also to neurodegenerative diseases, which are considered an emerging problem because of people aging.

## 6. Limitations and future research

The main limitation of this study is the lack of an economic evaluation of the H&S network, which could indicate whether the model provides cost savings for both local ASPs and RHS. Unfortunately, we do not have the economic information needed to carry it out. However, the H&P network has improved the existing health care supply and the ASPs located in Trapani and Palermo have already included the centers within their territorial network. Furthermore, some limitations could result from the fact that the study was carried out exclusively on inpatient administrative databases, without evaluating medical registers and patient satisfaction with the new organizational model implemented.

HS model could provide a means to better deal with the long-term pandemic sequelae. Indeed, neurological complications after SARS-CoV-2 infection (COVID-19) affecting the nervous system with associated muscular diseases have been reported (30, 31). COVID-19 related neurological symptoms are not limited to the motor (e.g., following stroke and encephalitis induced by the SARS-Cov2-infection) or the cardiopulmonary levels, but also include dysphagia, dysexecutive syndrome, apraxia, cognitive impairment, and psychiatric disorders such as depression, anxiety, and post-traumatic stress disorder, among others (32, 33). Thus, different organizational models were adopted in neurorehabilitation during the COVID-19 pandemic impacting the therapies time frame, the physical and mental health of healthcare professionals and the caregiver’s workload. There is still uncertainty about the effectiveness of these new therapeutic strategies on the management of neurorehabilitation services and future studies should explore the effect on the patients’ needs (34). Hence, this study could be extended in the future to examine the effects of the HS network considering a longer follow-up period.

Another important limitation is that we did not assess/investigate patients’ functional outcomes at discharge from the spoke centers. Then, we are not able to state (but only suppose) if and to extent neurological patients were more likely to be independent with functional ambulation, self-care, activity of daily life, etc. Indeed, given that in our HB we have used different innovative technology that are known to further potentiate clinical outcomes, it is conceivable that people receiving this treatment could have better results than those treated with usual territorial care.

## 7. Conclusion

According to this study, the activation of a HS network for intensive neuro-rehabilitation has facilitated the management of neurological patients, avoiding unnecessary migrations to other provinces and/or regions, and improving the regional care service for neuro-rehabilitation. Future research will be direct to investigate this aspect, focusing on before-after variations in hospitalization rates and origin–destination patient flows. Finally, it would be beneficial to reinforce the role of the GP as a case manager, as well as train citizens in disease management at their own home.

## Data availability statement

The data analyzed in this study were obtained from the Department of Health of the Sicilian Region, under the restrictions of non-public dissemination. Requests for access to these datasets should be addressed to Maria Cristina De Cola, mariacristina.decola@irccsme.it.

## Ethics statement

Ethical review and approval was not required for the study on human participants in accordance with the local legislation and institutional requirements.

## Author contributions

MCDC and AI: conceptualization, methodology, and writing – original draft preparation. RSC: validation and writing – review and editing. MCDC: formal analysis and data curation. VLB: investigation. AQ: supervision. All authors contributed to the article and approved the submitted version.

## Conflict of interest

The authors declare that the research was conducted in the absence of any commercial or financial relationships that could be construed as a potential conflict of interest.

## Publisher’s note

All claims expressed in this article are solely those of the authors and do not necessarily represent those of their affiliated organizations, or those of the publisher, the editors and the reviewers. Any product that may be evaluated in this article, or claim that may be made by its manufacturer, is not guaranteed or endorsed by the publisher.
